# Experiences and intentions of Ugandan household tuberculosis contacts receiving test results via text message: an exploratory study

**DOI:** 10.1186/s12889-020-8427-0

**Published:** 2020-03-12

**Authors:** Joseph M. Ggita, Anne Katahoire, Amanda J. Meyer, Elizabeth Nansubuga, Talemwa Nalugwa, Patricia Turimumahoro, Emmanuel Ochom, Irene Ayakaka, Jessica E. Haberer, Achilles Katamba, Mari Armstrong-Hough, J. Lucian Davis

**Affiliations:** 1 Uganda Tuberculosis Implementation Research Consortium, Makerere University, Kampala, Uganda; 2 Child Health and Development Centre, Makerere University, Kampala, Uganda; 3 Department of Epidemiology of Microbial Diseases, Yale School of Public Health, New Haven, CT USA; 4 Department of Population Studies, Makerere University, Kampala, Uganda; 5 Department of Medicine, Massachusetts General Hospital Global Health, Harvard Medical School, Boston, MA USA; 6 Clinical Epidemiology Unit, Makerere University, Kampala, Uganda; 7 Department of Social & Behavioral Sciences and Department of Epidemiology, New York University School of Global Public Health, New York, NY USA; 8 Pulmonary, Critical Care, and Sleep Medicine Section, Yale School of Medicine, New Haven, CT USA

**Keywords:** mHealth, Tuberculosis, Text message, Mobile phone, Community health worker

## Abstract

**Background:**

The World Health Organization (WHO) recommends household contact investigation for tuberculosis (TB) in high-burden countries. However, household contacts who complete evaluation for TB during contact investigation may have difficulty accessing their test results. Use of automated short-messaging services (SMS) to deliver test results could improve TB status awareness and linkage to care. We sought to explore how household contacts experience test results delivered via SMS, and how these experiences influence follow-up intentions.

**Methods:**

We conducted semi-structured interviews with household contacts who participated in a randomized controlled trial evaluating home sputum collection and delivery of TB results via SMS (Pan-African Clinical Trials Registry #201509000877140). We asked about feelings, beliefs, decisions, and behaviors in response to the SMS results. We analyzed the content and emerging themes in relation to the Theory of Planned Behavior.

**Results:**

We interviewed and achieved thematic saturation with ten household contacts. Nine received TB-negative results and one a TB-positive result. Household contacts reported relief upon receiving SMS confirming their TB status, but also said they lacked confidence in the results delivered by SMS. Some worried that negative results were incorrect until they spoke to a lay health worker (LHW). Household contacts said their long-term intentions to request help or seek care were influenced by perceived consequences of not observing the LHW’s instructions related to the SMS and follow-up procedures; beliefs about the curability of TB; anticipated support from LHWs; and perceived barriers to responding to an SMS request for further evaluation.

**Conclusion:**

Household contacts experienced relief when they received results. However, they were less confident about results delivered via SMS than results delivered by LHWs. Delivery of results by SMS should complement continued interaction with LHWs, not replace them.

## Background

Tuberculosis (TB) is the ninth leading cause of death worldwide [1]. To increase timely treatment and reduce transmission, the World Health Organization (WHO) recommends household contact investigation for TB in high-burden countries like Uganda [2]. During household contact investigation, confirmed index patients (i.e., those with known TB disease) are approached and a request is made to visit their home with the intention of identifying undiagnosed TB cases among their household contacts. The proportion of household contacts who complete TB evaluation in health facilities is low; this is the major obstacle to effective TB case finding in households [3, 4]. Moreover, household contacts who complete evaluation for TB during household contact investigation may have difficulty accessing the results of their evaluation [3].

To address these gaps, the WHO encourages public health implementers and researchers to use technology to create innovative approaches to reduce the burden of TB [5]. Globally, mobile health (mHealth) technologies have been used to improve care for a variety of illnesses, such as HIV, and to improve adherence to treatment [6, 7]. As such, mHealth technologies like SMS text messaging and electronic health records are thought to have significant potential for addressing challenges to ending TB [8]. Furthermore, many studies document the acceptability of text messaging for health-related communication, providing hope that SMS could improve communication between patients and providers and reduce the overall burden of TB on the health infrastructure [6, 9, 10]. However, most studies analyze text messaging as a means of improving adherence after an individual has already engaged with the health system. This overlooks a second important function text messaging could serve: to improve health awareness and facilitate care-seeking among individuals who are not already in treatment. Use of automated short-messaging services (SMS) to deliver results could improve TB status awareness and linkage to care [11]. Yet, little is known about how individuals who receive results via SMS decide to link to care.

As part of a prospective cluster-randomized trial of a text-messaging intervention to promote uptake of TB evaluation and treatment services in households, we carried out a qualitative evaluation of processes and contextual factors to understand participant experiences. We then used the Theory of Planned Behavior (TPB) as a conceptual framework to explore how attitudes, subjective norms, and perceptions of behavioral control influenced the intention to respond to an SMS. Specifically, we explored how household contacts receiving results via SMS influences follow-up intentions. The TPB has been widely applied to understand the behavior of patients [12, 13]. It theorizes that attitudes, subjective norms, and perceived behavioral control help form behavioral intentions, which are proximal causes of behavior [14]. Belief about whether performing a certain behavior will lead to a positive or negative outcome influences the attitudes toward the behavior, as do subjective norms, which reflect the individual’s perception of the expectations of others in his or her community regarding whether he or she should perform a given behavior. According to the TPB, intentions directly affect behavior, while attitudes and subjective norms influence behavior indirectly through their effects on intentions [14]. In contrast, perceived behavioral control affects behavior both directly and indirectly through intentions.

## Methods

### Study setting and design

Uganda ranks seventh among the thirty countries with the highest burden of TB/HIV [1], with a TB prevalence of 253 per 100,000 [15]. Kampala, the capital city, has an estimated population of 1.5 million people and accounts for a quarter of Uganda’s annual TB notifications. The Kampala Capital City Authority (KCCA) provides free primary health care, including evaluation and treatment for TB and HIV, at facilities throughout Kampala. This study took place at six primary health facilities and one general hospital overseen by the KCCA administration. All seven sites offer TB diagnostic and treatment services to residents of greater Kampala in specialized TB units. Each had implemented contact investigation as part of a program led by the National TB and Leprosy Programme (NTLP) in partnership with a non-governmental organization. Lay health workers (LHWs) based at KCCA facilities visit the homes of TB patients, screen household members for symptoms of TB, and refer symptomatic household contacts to health centers for TB evaluation.

In this setting, we carried out cross-sectional, qualitative interviews to explore the experiences and intentions of household contacts after they had received SMS messages containing results of laboratory testing for TB. We conducted semi-structured interviews with household contacts of TB patients who had received SMS messages as part of a randomized, controlled trial called the Mobile Health for Implementation of Home-Based TB Contact Investigation (mHealth CI) Study (Pan-African Clinical Trials Registry #201509000877140). We used the TPB to develop the interview guide and inform the analysis.

### Characteristics of the home-based mHealth intervention and SMS messages

In accordance with contact investigation guidelines from the Uganda NTLP [16] and WHO [2], LHWs visited the homes of index patients to identify household contacts. We defined household contacts as individuals who had spent one or more days or nights sleeping under the same roof as an individual diagnosed with microbiologically confirmed pulmonary TB [17]. LHWs then screened household contacts for TB symptoms and clinical characteristics associated with rapid progression to active TB (i.e.*,* living with HIV, age < 5 years). For individuals screening positive, LHWs either collected and transported sputum to facility-based laboratories for analysis (intervention arm, individuals aged ≥5 years only) or referred them for follow-up TB evaluation at a health facility (control arm). All children aged < 5 years from both arms were referred for follow-up TB evaluation at a health facility. As part of the mHealth CI trial, household contacts in the intervention arm received their diagnostic results via SMS. The results of the main study have been reported elsewhere [18].

The SMS intervention in the trial was designed to provide the results of testing, instructions about additional evaluation or treatment, and reminders to come to the clinic for evaluation as needed (Table 1). These messages were sent to household contacts based on their TB status. Household contacts with confirmed TB were asked to proceed to the health facility for treatment. Household contacts for whom TB was ruled unlikely were told that their tests did not show TB and were advised to respond “HELP” if their symptoms did not improve.

**Table 1:** Interview sample

Sex	Age	Education	Occupation	Individual Income	Type of SMS Received	Action Requested of Household Contact	Action Taken By Household Contact	Quotation
Female	18	Secondary	Student	0 UGX	Unlikely TB	Reply HELP if you do not get better	None	My parent showed me the message and I was happy that I did not have TB
Female	19	Secondary	Student	0 UGX	Unlikely TB	Reply HELP if you do not get better	None	My mother told me and I asked her to see the message and felt good
Female	22	Primary	Unemployed	0 UGX	Unlikely TB	Reply HELP if you do not get better	Waited for the LHW to come for a follow-up visit	Well, it is okay if she wants to send through a message, I will still wait for her to come and I ask her
Female	22	Secondary	Unemployed	0 UGX	Unlikely TB^b^	Reply HELP if you do not get better	None	I just felt happy because I wanted to know the actual diagnosis.
Female	40	Primary	Self-employed	50,000 UGX	Unlikely TB	Reply HELP if you do not get better	None	I got relieved and provided support to the patient
Female	61	Primary	Unemployed	0 UGX	Unlikely TB	Reply HELP if you do not get better	Waited for the LHW to come for a follow-up visit	I was happy but also wanted to talk to her the next time she visits
Male	19	Secondary	Unemployed	0 UGX	Unlikely TB	Reply HELP if you do not get better	Sent “HELP”	On that day it was sent to me I replied with HELP to it because I felt the testing machine might have had an error
Male	29	Tertiary	Self-employed	1,000,000 UGX	Unlikely TB	Reply HELP if you do not get better	Visited a clinic at a later date	…I expect to go to South Africa for about 3 months and we were advised to go for a check-up for TB and HIV and the results were needed and that is why I had to go for that test.
Male	37	Secondary	Self-employed	350,000 UGX	Confirmed TB^a^	Visit a health centre for treatment	Telephoned the LHW to schedule an appointment	I immediately contacted the LHW, I called her and we scheduled the appointment the next morning
Male	39	Tertiary	Self-employed	800,000 UGX	Unlikely TB	Reply HELP if you do not get better	Waited for TB longitudinal message	I waited for a second message but it hasn’t come through

Abbreviations:

Legend: The text of the English version of the SMS messages were as follows:

^a^Confirmed TB: “(Participant name) your tests show TB. (Participant name) please come to (health centre name) for TB treatment if you have not already.”

^b^Unlikely TB: “(Participant name) your tests do not show TB, but if you do not get better, reply HELP (no charges apply). Tests can miss TB if done too early.”

### Study population, sampling, and recruitment

We identified household contacts from the intervention arm of the mHealth CI trial. Household contacts were eligible for selection if they had been sent results via SMS and confirmed as having received the SMS during the quantitative phase of this mixed-methods study, which has been published separately [19]. We purposively sampled household contacts to ensure representation by gender and a range of ages. If the selected participant was under the age of 15 years, we interviewed their parent or guardian. We planned an interim analysis after every ten participants had been interviewed and to end recruitment upon reaching thematic saturation. We defined saturation by repeated emergence of themes related to how people felt about receiving results via SMS, independent of what the results were. We chose to do that because it has been found out that for most studies with clearly defined aims, only 10 to 15 interviews per subject group are required to reach saturation and in rare cases among homogenous populations as few as 6 interviews per subject group are required for content validity [20].

### Procedures

The LHW, known to the participant, invited a household contact for a face-to-face interview at home or any public place of his or her choosing. The LHW then accompanied the researcher (J.G.) to the interview venue to facilitate introductions, and then left prior to the interview. The researcher (J.G.) was a male, bachelor’s-qualified social scientist with 3 years’ experience facilitating qualitative data collection and fluency in both Luganda and English. He interviewed all participants using a semi-structured interview guide (Additional file 1: Appendix S1). The interview guide was designed in English, translated into Luganda and back translated to ensure the meaning was not altered. A female researcher (T.N.), with prior experience conducting qualitative research, attended all sessions to take notes and assist in asking follow-up questions. The interview guide sought to elicit narratives from participants about their feelings and behaviors following receipt of a TB-related SMS message, their intended actions following receipt of the SMS instructions, and the social and psychological antecedents of these intentions. Interviews were conducted in English, Luganda, or in both languages, in a private setting chosen by the participant. The interviewer also recorded basic demographic information, including age, sex, education level, occupation, and income. All interviews were audio-recorded with each participant’s permission.

Immediately following each interview, the interviewer compiled a debriefing report, which was discussed with the note taker. We used the debriefing report to identify topics requiring more probing in subsequent interviews. All interviews were transcribed, verified, translated into English if necessary, and imported into ATLAS.ti 8.1 [21] for analysis.

### Analysis

The lead coder (J.G.) read all transcripts to identify meaningful analytical segments. We developed codes using an inductive approach to content analysis [22] during the first stage of analysis. We then discussed the emerging categories with other members of the research team, and finally applied codes to all transcripts. We analyzed the codes and emerging themes in relation to the Theory of Planned Behavior.

### Protection of human subjects

Each participant provided written informed consent for the interviews as part of the parent trial. The School of Medicine Research Ethics Committee at the Makerere College of Health Sciences (2013–061), the Uganda National Council for Science and Technology (IS 104), and the Human Investigation Committee at Yale University approved the study (1505015812).

## Results

### Characteristics of participants

In October and November 2017, LHWs telephoned ten household contacts 10 to 16 weeks after receiving test results via SMS to recruit them for a face-to-face interview. All ten participants accepted, including six women and four men. Median age was 26 years, ranging from 18 to 61 years; other characteristics are provided in Table 1. One respondent confirmed receiving positive TB testing results via SMS; the remainder confirmed receiving negative results. Interviews lasted an average of 33 min, ranging from 30 to 45 min.

Several major themes emerged from the interviews. Participants reported experiencing anxiety about a possible diagnosis of TB, relief upon confirming TB status via SMS, and ambivalence about SMS as a medium for communicating results. Participants also described their actions after receiving test results, and how these actions influenced their perceptions of the consequences of not observing the LHW’s instructions related to the SMS and follow up procedures; beliefs about the curability of TB; anticipated support from LHWs; and perceived barriers to responding to an SMS request for further evaluation.

### Household contacts’ experiences before and after receiving TB test results

#### Anxiety about a possible diagnosis of TB prior to receiving the SMS results

Household contacts reported feeling anxious about the outcome of TB evaluation prior to receiving the SMS. One young woman said she knew she was living with a TB patient and had been told by the LHW that she had symptoms suggestive of TB. She continued:

“*Now, since you have some signs showing that you have TB and you are very eager to know whether these signs are of TB… Now, do I have TB or I don’t have? You can be so anxious to know…What are the results*?” [Female, 18 years]

The TB education that household contacts received from LHWs during household contact investigation increased their concerns about TB. Most indicated that the perceived consequences of the test results contributed to their anxiety:

“*The question I had: ‘If the results come out and show that I am infected, what will I do?’ You know, I fear taking tablets so much and I kept asking myself what I will do and where to start from. My husband had explained to me that in case I don’t take the medicine well then I might be put on injections and whenever I looked at my child I felt she is still young that needs my support and I had made up my mind to take the medication in case I am found positive*.” [Female, 22 years]

Most household contacts who reported anxiety also said that, during the time between the household contact investigation visit and receipt of their test results via SMS, they believed they had TB infection and urgently wished to confirm their status.

#### Relief upon confirming TB status

Several household contacts reported relief upon learning their TB status via SMS, including the parent of one participant with TB and several without TB. Those with a negative TB diagnosis had unanimously assumed they would receive a positive TB result because they were staying with a TB patient and had a cough. For example, a 29-year-old man described his experience receiving the SMS:

“*You know, the tension I had knowing that my sister is infected… I thought that even the rest of us at home might be infected with the same disease. It helped me. In fact, the time the message came in I was doing my work that side of Mubende. It came at the time I didn’t expect it, it was just like the same way any normal message would come in, you might not be aware of what has come through. I later opened the message and it was indicating that I was TB-negative, and I felt relieved of that burden that I had on mind.*” [Male, 29 years]

Notably, the respondent who received an SMS confirming a positive diagnosis for his young daughter also described a sense of relief. When he received the result via SMS he explained:

“*At that time, I felt so relieved. I had spent time buying different types of medicine but the child would still continue coughing. On that day I felt relieved because I knew I had come to the right diagnosis of the illness. I got to know the type of medication required and the disease she is suffering from*.” [Male, 37 years]

For this respondent, even a positive TB result provided relief because it ended prolonged uncertainty over the source of the illness. However, several household contacts who received negative TB results via SMS said that, while they felt relief, they also desired further evaluation for their ongoing symptoms in order to confirm a specific diagnosis and obtain appropriate treatment.

#### Ambivalence about SMS as a medium for communicating test results

Household contacts expressed ambivalence about SMS as a medium for communicating TB results. On one hand, participants said they appreciated the accessibility and convenience of receiving results via SMS. Convenience was described as being able to continue daily activities while waiting for results, not having to travel, and receiving results in a timely fashion. One man who received a negative result explained,

“*…it was easier for me because I had my phone and at any time unexpectedly you receive a message and get to know at that particular time your status as opposed to inconveniencing someone by asking them to go back to the hospital. You might not be infected with TB and then you incur costs of transport*” [Male, 29 years]

On the other hand, the same household contacts who praised the convenience of SMS also said that they remained concerned about their TB status even after receiving the test results via SMS because they were not fully confident that the results were accurate. They cited a belief that new technologies may be error-prone as an explanation of their uncertainty about their SMS-delivered results. Some reported a preference for face-to-face delivery of results:

“*I do not support it. I do not support sending results through a message. I would love to be called on phone or actually a health worker coming and talk to you at home because you might again doubt whether you are talking to the right health worker that took your sample*.” [Female, 22 years]

Similarly, others noted that they hoped for follow-up interactions with LHWs to explain results more fully and allay concerns. These sentiments were emphasized by a young woman who was still hoping to talk to the LHW:

“*I didn’t feel bad because the results were showing that I didn’t have TB… but still I wanted to talk to the LHW. I decided to accept the results but still wait for the LHW to tell me more*.” [Female, 22 years]

This participant said that her first inclination was to call the LHW for clarification. However, her husband stopped her; he felt that LHWs are busy and should not be bothered with telephone calls. Nonetheless, they expected a follow-up visit from the LHW.

### Actions of household contacts after receiving a test result

The man who received a positive TB test result on behalf of his child was asked to go to the health center for TB treatment. He immediately called the LHW to schedule a visit to the clinic. Household contacts who received negative results were asked to reply “HELP” if they did not get better. These household contacts responded in range of ways; some replied “HELP”, while others decided to wait and do nothing (Table 1):

“*I decided to wait for her to come and explain to me because truthfully speaking I thought I had TB since I was staying close to my husband for a long time….”* [Female, 22 years]

A woman who received a message explaining that she had tested negative for TB said that she decided to wait at home to avoid bothering the LHW with frequent phone calls. She described how the household had a strong relationship with LHW and her expectation that they would meet again in person, at which time she would have the opportunity to ask for more explanation.

### Contributors to household contacts’ intention to respond to SMS-suggested actions

**Fig. 1:**
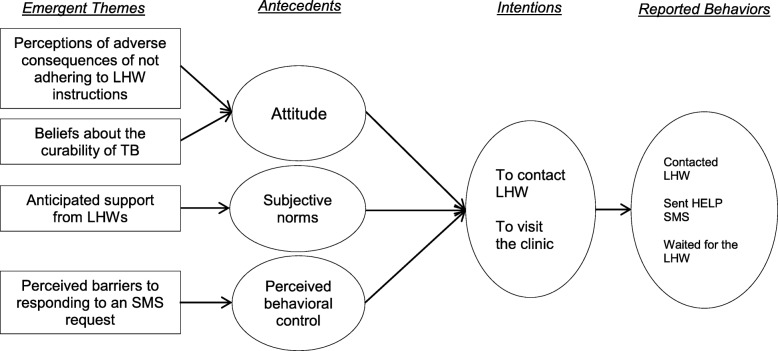
Conceptual model of contributing factors to behaviors of household contacts based on the Theory of Planned Behavior. The figure presents a conceptual model of contributing factors to behaviors of household contacts after receiving TB test results via SMS during household contact investigation. Contributing factors are categorized and linked to participants’ stated intentions and actual behaviors

We use the TPB framework to organize the factors that household contacts cite as contributing to their intention to respond with the actions suggested in the SMS (Fig. 1). Participants generally had favorable attitudes toward responding to the SMS as a result of either outcome of their evaluation; they believed that, if treated, they would be cured. Similarly, they believed that if they followed the LHWs’ instructions related to the SMS, they would receive support to access treatment or else they would miss access to treatment. Participants were influenced by their belief that LHWs would provide support to the household in the future, which contributed to their intention to visit the clinic or contact a LHW to access treatment. However, some participants described a lack of perceived behavioral control; they felt that they did not have the resources to perform the required follow-up behaviors. We describe these themes in detail below.

#### Perceptions of adverse consequences of not adhering to the LHW’s instructions

Perceptions of adverse consequences of not observing the LHW’s instructions encouraged household contacts to either reply to the message with “HELP” or call the LHW for a scheduled visit. Having been directed to respond, participants believed that following the instructions of the LHW was a prerequisite for accessing care and treatment.

*“It seemed that in case you do not respond to the message they might not be able to help you. I was tested and the results were sent to me and I was requested to reply to it. So, I had to do it because I knew it means a lot when it comes to receiving treatment.”* [Male, 37 years]

This man believed that he could access TB care and treatment for his child only if he acted on the instructions from the LHW.

*“One day it was sent to me and I replied to it and she immediately called back and said that she was going to come…I had been told that in case I replied they would provide support immediately”* [Male, 19 years]

This young man was confident that he would achieve access to further evaluation if he replied to the message as directed.

#### Beliefs about the curability of TB

The belief that TB is curable motivated household contacts to perform follow-up behaviors, regardless of whether the result that they received was positive or negative. The parent of the child who was diagnosed with TB mentioned that he had been told that medication cures TB and had seen the treatment work for one of his brothers. He highlighted his need to see his child receive the right medication and get cured.

“*We had already been told that TB heals and medication is free, that we only need to be committed in taking it; not to miss and take it at the right time. I knew that at least my child will be able to take the right medication as opposed to taking wrong treatment for a long time without feeling fine or getting better*.” [Male, 37 years]

The father’s expectation that his child could be cured motivated him to contact the LHW. Another household contact who received a negative TB result message and replied “HELP” reported feeling unwell and wanting to confirm his status and access treatment.

*“Since I was still feeling unwell I sent HELP. I said to myself: I need to know so that in case I need to get treatment I start right away when it is still early enough and get cured… The LHW called me and promised to come, which she did and decided to refer me to the clinic.”* [Male, 19 years]

#### Anticipated support from lay health workers

Anticipated support from the lay health workers had a positive influence on participants’ intentions to visit a health facility or reply to the SMS if symptoms developed or persisted. Participants felt that the support would allow them to bypass the waiting areas at the clinics and avoid delays in service:

*“…the LHW had told me that she would help me in case I got there. It won’t require me to make line or anything; it would be just like an appointment.”* [Male, 19 years]

One participant explained that he had developed trust in the LHW’s promise to see them through the clinic procedures following the home visit and other face-to-face interactions:

*“Since I had received some counselling during the LHW visit…I found it easy to follow what I was told. I had earlier talked to the LHW again face to face.”* [Male, 37 years]

Participants reported that their intentions to return to the clinic or request help in the future were premised on their belief that the LHW would support them if they did so.

#### Perceived barriers to responding to an SMS request for further evaluation

The child who received positive results went to the clinic and initiated treatment for TB. The nine participants who received negative results felt that if they had been asked to return to the clinic, they might have found it difficult to comply. Participants said that clinic hours conflicted with their work schedules:

*“If I had been requested to go for further evaluation I would have failed since I was away upcountry and the results had been sent from Naguru clinic, which could inconvenience my working time. It would inconvenience my other duties…”* [Male, 29 years]

Several other household contacts also imagined that the distance to the clinic and busy working schedules would interfere with their intention to complete TB evaluation.

## Discussion

Many household contacts who complete screening for TB never complete their evaluation or receive their test results [3]. These losses of possible TB patients to follow-up are a major hindrance to delivery of timely community-based TB treatment and prevention. Automated SMS have been proposed to reduce barriers to receiving diagnoses and linking to care [9, 23]. However, little is known about how this approach to communicating TB results may influence patients’ linkage to care, especially during community-based active TB case finding. We found that SMS is a useful tool for delivering TB test results but cannot replace post-test counseling interactions between the health worker and the household contact. It is these personal interactions that give household contacts confidence in test results, help relieve their anxiety about testing, and motivate them to respond as requested.

We found that most participants described SMS as highly acceptable and more convenient than face-to-face communication of results. This finding aligns with reports that SMS-delivered test results are acceptable in principle among people who have not experienced it firsthand [9, 24]. In our study, however, participants cautioned that this mode of communication limited their interactions with health workers; without this interaction, they worried that their negative results might be inaccurate. In contrast, others have reported that text messaging of laboratory results can improve communication and overall clinical care by quickly notifying patients of their status [25].

We also found that household contacts who received results via SMS intended to follow the SMS-delivered suggestions because they knew that TB could be cured with treatment, and because they expected to receive treatment support from LHWs. A study of SMS technology to improve TB treatment adherence in Peru similarly found that awareness of the seriousness of TB and fear of death were powerful motivators to adhere to treatment among TB patients [7]. Similarly, we found that among individuals who are household contacts of TB patients and undergoing evaluation for TB, beliefs about the seriousness and curability of TB also shape their intention to follow up at the clinic if their own symptoms persist or return. This finding is important because household contacts of TB patients have a continuing risk of progressing to active TB within 2 years after the initial exposure [26]. While we do not have longitudinal data to explore subsequent health-seeking behavior, according to the TPB, behavioral intentions such as these can be an important predictor of future care-seeking behavior [12]. In our study, some household contacts lacked intention and many did not follow-up which is undesirable from a public health perspective.

Finally, like others, we identified clinic hours that conflict with working schedules as a potential barrier to linkage to care [24, 27]. Participants anticipated that misalignment of clinic hours with their work schedules would prevent them from visiting the clinic if this kind of visit became necessary. However, Chadha et al. [11] reported that 51% of TB patients attend the clinic within 7 days of receiving their TB diagnosis via a text message, suggesting that this perceived barrier may become less important when patients are motivated by a TB diagnosis. Others have reported that, although household contacts fault inconvenient hours as a barrier, they still felt that counseling from LWHs might be reducing the burden of this barrier [27].

Our study has some limitations. While our sampling strategy was based on reaching saturation for factors relevant to the intention to respond to the SMS, our analysis is limited by the absence of adult household contacts who received a positive TB diagnosis. Only one person received SMS indicating positive result; moreover, the person who received a positive result was a child. Because we interviewed only one person who received SMS for a positive result, we may not have achieved saturation among this subgroup. Second, we were unable to link TPB antecedents to participants’ subsequent behaviors, which would have enabled evaluation of the actual impact of the SMS. Third, we did not interview contacts of index patients with drug-resistant TB. It is not possible ascertain whether these findings are relevant to contacts of index patients of different classifications of TB.

The study also had some strengths. Although a number of commercial software systems have been developed and implemented in sub-Saharan Africa to deliver test results via SMS, especially in the area of new molecular diagnostics, literature on use of SMS to deliver diagnostic results is limited, especially for TB laboratory results. We provide a foundation for understanding how household contacts of TB patients experience receiving results via SMS and form intentions to link to care, enabling us to make preliminary recommendations to researchers designing SMS interventions. Our findings from this exploratory study can be used to develop surveys to understand the phenomenon in a more generalizable way.

## Conclusion

Household contacts may feel relieved of their anxiety when they receive their TB diagnostic results via SMS, but they are less confident about results delivered via SMS than about results delivered by LHWs. Delivery of results by SMS should complement continued interaction with LHWs, not replace it.

## Supplementary information

**Additional file 1 Appendix S1:** Interview guide. **Appendix S2:** Demographic Information Sheet.

## Data Availability

All de-identified transcripts are available from the corresponding author upon request.

## Abbreviations

CI: Contact Investigation
HIV: Human Immunodeficiency Virus
KCCA: Kampala Capital City Authority
LHW: Lay Health Worker
mHealth: Mobile Health
NTLP: National Tuberculosis and Leprosy Programme
SMS: Short-messaging Services
TB: Tuberculosis
TPB: Theory of Planned Behavior
WHO: World Health Organization
