# Supplementary material for: Experiences and intentions of Ugandan household tuberculosis contacts receiving test results via text message: an exploratory study
Source: BMC Public Health. 2020 Mar 12;20:310. doi: 10.1186/s12889-020-8427-0 (PMC7068887; doi:10.1186/s12889-020-8427-0)
Supplement: Supplementary file 1 — Additional file 1 Appendix S1: Interview guide. Appendix S2: Demographic Information Sheet. [file 12889_2020_8427_MOESM1_ESM.docx]

**Online supplement**

**Appendix S1: Interview guide**

Thank you for joining this discussion. My name is ___________ and I am a researcher from ________. I would like to get your experiences with TB text messaging. You have been chosen to participate in this interview because you participated in a Contact Investigation study and received a TB text message sent from ________health centre. I would like to learn about your experience towards an SMS showing your health information, the response you made after receiving the text message and the factors that influenced that response. This will help us learn more about sending TB related health information to household contacts of TB patients.

There are no right or wrong answers. Feel free to share your views as they are. If you accept, I will be recording this interview to make sure that I do not miss out any important information. Remember that all this information will be kept confidential and I will not use your name or any identifiable information in connection to what you will say.

Should we continue with this interview? Do you allow to be audio recorded?

- To begin with, what do people normally use their phones for?
  - How do you use your phone and for what purposes?
  - How do you normally feel about sending and receiving short messages (SMS)? In what ways do you use SMS in your daily life? From whom do you receive SMS and to whom do you send an SMS?
- Tell me about receiving an SMS showing your TB results?
  - Were you able to read it?
  - If NO, why? If YES, What was it saying? Was it just the results or were you given additional information?
  - What thoughts and questions came to your mind when the idea that your TB results would be sent to you via an SMS was introduced to you?
  - What were some of your concerns?
  - What were some of the issues that you felt relieved about given the possibility of receiving your results via SMS?
  - Whom did you discuss this idea with if at all and what were their thoughts?
- Once the results were sent and you saw the notification on your phone what thoughts came to your mind? How long did it take you to open the message? What did you feel when you read it?
- What were the instructions like? What did you do with the message once you had read it? Why?
  - Was it easy for you to follow what a TB text message from the health facility said and why?
- What influenced your decision of responding [or not] to an SMS sent from the clinic?
  - Influence from the family members and friends [Positive and Negative]
- What do you regard as important in helping you respond to an SMS sent from the
- clinic?
- Concluding statements
  - Are there any other issues that come to mind when you think about communicating with health facilities about TB testing results by text messaging?
  - Is there anything else we haven’t talked about that you would like to tell me?

Thank you for your time.

**Appendix S2: Demographic Information Sheet**

1. Gender
   1. Male
   2. Female
2. Highest level of education
   1. None
   2. Primary School
   3. O-level
   4. A-level
   5. Tertiary education
   6. Other (specify)_________________
3. Occupation
   1. Self-employed
   2. Employed
   3. No employment
   4. Housewife
   5. Student
   6. Other (specify)_________________
4. Marital status
   1. Married
   2. Not married (Never married before)
   3. Not married (Separated/Divorced)
   4. I do not know
   5. Not willing to share that
   6. Other (specify)_________________
5. Are you the owner of the phone on which you received the TB text message?
   1. Yes
   2. No
6. If No, who owns the phone? ______________________
